# Comment on Pu et al. Predicting Postoperative Lung Cancer Recurrence and Survival Using Cox Proportional Hazards Regression and Machine Learning. *Cancers* 2025, *17*, 33

**DOI:** 10.3390/cancers17040697

**Published:** 2025-02-19

**Authors:** Taro Okura, Ken-ichiro Sakata, Tatsuki Itagaki

**Affiliations:** Oral Diagnosis and Medicine, Faculty of Dental Medicine, Graduate School of Dental Medicine, Hokkaido University, Kita-13 Nishi-7, Kita-ku, Sapporo 060-8586, Japan; gogotaro@den.hokudai.ac.jp (T.O.); sakata-0303@den.hokudai.ac.jp (K.-i.S.)

Pu et al. published an interesting paper entitled “Predicting Postoperative Lung Cancer Recurrence and Survival Using Cox Proportional Hazards Regression and Machine Learning” [1]. In the study [1], Pu et al. conducted a detailed survey and performed various analyses. However, there is room for improvement regarding analysis. As dentists, we often provide oral care for patients with lung cancer, so prognosis prediction is important to us as well. We would like to present our comment from the perspective of colleagues. 

Regarding the selection of variables for multivariate analysis, they selected those that were significant by univariate analysis. This method is no different from a mechanical selection method. As for machine learning, cross-validation was performed, but the sample size of the test set was 60 persons, and the number of persons used in the training set was 240 persons. The number of people used in each set appears to be minimal, but more data would be preferable [2]. For the reasons stated above, therefore, we as experts should select existing risks or potential risks as variables in multivariate analysis.

In addition, regarding the predictive model for lung cancer recurrence 2 or 5 years after surgical treatment, because the Cox model and machine learning models with a small dataset are nonparametric methods, they can only predict with respect to their own data, and because of the lack of extrapolation, the models have little validity [2]. On the other hand, logistic regression analysis is used to predict recurrence, and in this case, the predictive model for lung cancer recurrence 2 or 5 years after surgical treatment has some validity. A parametric method using functions such as exponential functions would not only provide extrapolation and allow some predictions beyond 5 years postoperatively but would also allow the estimation of the mean time to postoperative recurrence. Parametric methods are more general, less susceptible to individual data, and more capable of extrapolation. Therefore, we recommend a parametric method with an exponential function. 

In conclusion, although their study is useful, existing parametric methods are sufficient to achieve their aims with respect to predicting postoperative recurrence without the use of machine learning. If machine learning is to be used, it should be used as they said in a condition with a huge sample size.
